# Comparison of translational and rotational modes towards passive rheology of the cytoplasm of MCF-7 cells using optical tweezers

**DOI:** 10.3389/fphy.2022.1099958

**Published:** 2023-01-09

**Authors:** Srestha Roy, Rahul Vaippully, Muruga Lokesh, Gokul Nalupurackal, Privita Edwina, Saumendra Bajpai, Basudev Roy

**Affiliations:** 1 Department of Physics, Indian Institute of Technology Madras, Chennai, India; 2 Department of Applied Mechanics, Indian Institute of Technology Madras, Chennai, India

**Keywords:** viscosity ratio, relaxation time, cytosol, translation, rotation, optical tweezers, pitch rotation, thermal fluctuation

## Abstract

A colloidal particle placed inside the cell cytoplasm is enmeshed within a network of cytoskeletal fibres immersed in the cytosolic fluid. The translational mode is believed to yield different rheological parameters than the rotational mode, given that these modes stretch the fibers differently. We compare the parameters for Michigan Cancer Foundation-7 (MCF-7) cells in this manuscript and find that the results are well comparable to each other. At low values of 0 Hz viscosity, the rotational and translational viscoelasticity matches well. However, discrepancies appear at higher values which may indicate that the cytoskeletal modes involved in rotation and translation of the particle are getting invoked. We also show that the 0 Hz viscosity increases as the cell ages under the conditions of constant room temperature of 25^°^C on the sample chamber.

## 1 Introduction

At the cellular level, metabolism is considered as an enormous set of reactions between bio-molecules [1, 2]. These reactions avoid any prolonged disturbance in the cell that can lead to pathological changes, including death or systemic diseases [3, 4]. From a physical standpoint, a reaction can only occur if the reactants approach each other. In the equilibrium-state solution, Brownian motion or free diffusion is one of the sources of the movement of particles, and an increase of diffusion rate increases the probability of molecular encounters leading to the biochemical reactions. The cytoplasm is, however, a complex and crowded medium [5, 6], where diffusion of biomolecules is hindered, and thus diffusion can be treated as a factor limiting the reaction rates in the cell [7, 8]. Decrease of diffusion rates would decrease rates of metabolic reactions and eventually lead to cell damage [9].

Detecting the rheological parameters in the cell cytoplasm has proved challenging. Inside the cell, not only is the viscosity dependent upon probe particle size used to perform the rheology [10, 11], it also varies from intracellular location to location [5, 12]. The cytoplasm is comprised of the cytoskeleton and the cytosol. It has been shown that when a particle is moved while trapped inside the cell, the behaviour depends not only on the particle size but also the pulling speed [13]. Further, active motion inside the cell cytoplasm makes calibration difficult [14], since the amount of motion experienced by a tracer particle in the presence of the active motion would be higher and different than that due to passive thermal origin. In light of this, a technique exclusively utilizing the passive thermal motion of an optically confined particle to ascertain rheological parameters becomes complicated.

Early attempts at ascertaining the viscoelasticity of a cell searched for appropriate models to fit experimental data, like [15]. Recently a technique was developed where, the power spectral density of the translational motion of an optically trapped particle is used to ascertain the viscoelasticity of the cytoplasm [12, 16]. The active motion was believed to be till 10 Hz [17–19], above which the motion was assumed to be passive thermal motion, which was then fitted with an expression derived from the Generalised Maxwell Model or the Jeffery’s model of viscoelasticity [20–22]. It may be noted here that generally the active motion appears below 10 Hz in power spectral density for intracellular motion, but then there are reports of some cells exhibiting activity till 100 Hz [23]. However, it was shown in [12] that the assumption of active motion persisting till 10 Hz was a good approximation for MCF-7 cells, to which we focus our attention for the present manuscript. The model assumed that the viscosity depended upon frequency but that the 0 Hz viscosity was the entity that could be compared. It was found that the values for the 0 Hz intracellular viscosity matched well with that ascertained using Fluorescent Correlation Spectroscopy (FCS) [11] of fluorescent particles placed inside the cytoplasm where the probe size was about 1 *μ*m. It was shown by FCS that the viscosity increased till 100 nm radius of particle after which it became constant at $\frac{\eta_{eff\;}}{\eta_{water\;}}$ becoming about 13 [11]. In [11], it was shown that 1 nm radius probe shows a viscosity of about 2.5 times that of water, while a probe of 10 nm shows a viscosity of about 8 times that of water. The same intracellular fluid shows a viscosity of 30 times that of water, when probed with a 100 nm radius probe particle. This [11] also points to a situation where increasing the probe size from 100 nm towards higher values does not alter the value of the viscosity measured. Thus, should we do the experiment with particles of size 1,000 nm, we are going to get same values of viscosity as 100 nm probe size. The fits to the thermal PSD matched well with the viscosity for large particles from the FCS. Moreover, birefringent particles made from RM257 tend to lose birefringence for particles smaller than 600 nm diameter due to the energetics of the liquid crystalline droplets. Hence it was necessary to use 1 *μ*m particles.

The rheology can be performed both in the translation and in rotational modes. One of the advantages of probing rotational modes is that the probe is localised at one point while turning, thereby spanning less of the spatial variations inside the cell. This is a problem while using translational modes since the viscosity changes from location to location inside the cell, so that small changes of the position can introduce alterations in the viscosity.

It is this technique that we use here to compare translational motion and rotational motion. The rotational motion probes an entirely different mode of the cytoskeletal stretching than the translational motion [24–26]. We find that at low 0 Hz viscosity, which appears away from the nucleus mainly, the values for translation and rotation seem similar but at high values of viscosity, there seem to be discrepancies which could be a result of the cytoskeletal fibers being involved.

## 2 Materials and methods

**Figure 1:**
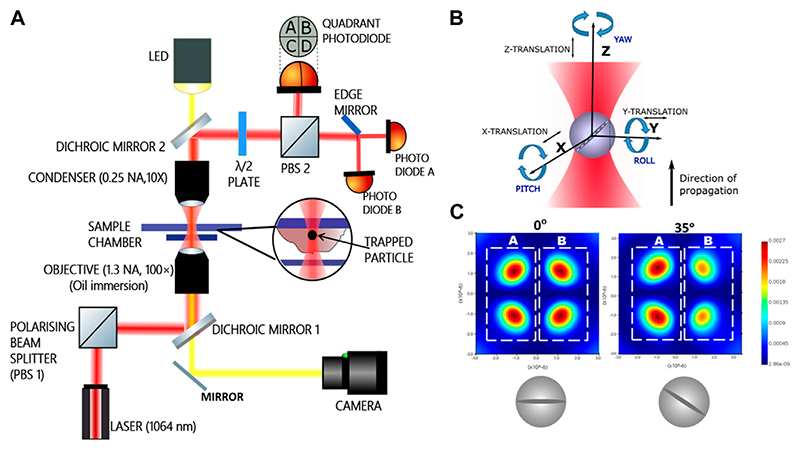
**(A)**Schematic diagram of the experimental set up. The dichroic mirrors are used to reflect the 1,064 nm beam from the laser to make it pass through the sample chamber where it is employed to trap and then to the quadrant photodiode and the pair of photodiodes. Cells fed with the particles are attached to the upper surface of the sample chamber. **(B)** The three rotations with respective nomenclatures **(C)** intensity changes in two halves of the simulated scatter pattern from the optically trapped birefringent sphere under crossed polarizers at two different pitch angles. The halves **(A)** and **(B)** are split and made incident on respective photodiodes.

The experimental set up comprises of an optical tweezers kit (Thorlabs, USA) in an inverted configuration. The schematic diagram of the apparatus is shown in Figure 1 while the sample for study is placed in the chamber. The cells are attached to the upper cover-slip (Blue Star, 75 mm length, 25 mm width, and 1.1 mm thick) with buffer while a square cover slip (Blue star, no. 1 size, English glass) is placed at the bottom to form the sample chamber. Visible light from an LED lamp placed above the sample chamber is transmitted through a dichroic mirror to illuminate the sample. After passing through the sample chamber it passes through another dichroic mirror and is directed to a CMOS camera *via* a reflecting mirror.

We use human breast cancer cells of MCF-7 cell line (obtained from National Center for Cell Science, Pune, India). The cells were maintained in Dulbecco’s Modified Eagle’s Media (DMEM) supplemented with 10% Fetal Bovine Serum (Gibco) and 1% penicillin-streptomycin (Gibco) to prevent contamination by any bacterial growth. For the experiment, the cells were grown on gelatin coated glass coverslips (50 mm × 20 mm). Initially, the glass coverslips were cleaned thoroughly in detergent followed by wash in concentrated HNO_3_. The coverslips were sterilized by UV treatment for 1 h. For gelatin coating, a droplet of .1% gelatin solution was added to the center of the coverslip and incubated at 37 C for 1 h. The coverslip is then washed using 1X PBS and 20 *μ*l of cell suspension (105 cells/ml) was added to the center of the coverslip. The cells were incubated at 37 C and 5% CO_2_ for 1 h to allow the cells to settle and attach to the surface. CO_2_ along with H_2_CO_3_ present in the nutrient medium assists in maintaining the pH level within the range of 7.2–7.4 (slightly alkaline) which is essential for keeping the cells alive. After the cells are attached, 500 *μ*l of fresh media is added. For probing the cytoskeletal rheology, 10 *μ*l of 1 *μ*m birefringent particles (5 *μ*l of stock suspended in 100 *μ*l of sterile serum free media) is added to the cells and incubated overnight. The cells were then washed with 1X PBS to remove the excess particles and fresh media is added.

The cells were incubated with birefringent liquid crystalline particles RM-257 (Merck) having diameter around 1 ± .2 *μ*m. To prepare RM-257 particles [27–29], 50 ml of 99% ethyl alcohol is heated to 55 C in a beaker placed on a hot plate and 150 ml of deionized water is heated in a beaker to 75 C on another hotplate simultaneously. The beakers containing the ethyl alcohol and deionized water are covered tightly with aluminium foils to prevent evaporation while heating. When the ethyl alcohol reaches 55 C, 40 mg of RM257 (1,4-Bis-[4-(3- acryloyloxypropyloxy)benzoyloxy]-2-methylbenzene) precursor powder is added to it and constantly stirred with a magnetic stirrer. After it is completely diluted, this mixture is added dropwise to the hot deionized water at 75 C. Then the mixture is left to evaporate until all the ethyl alcohol is evaporated leaving behind only the suspension of RM 257 particles in water. To suppress the evaporation rate, the beaker containing the mixture is covered with a perforated aluminium foil. Controlled evaporation rate by stirring at 150–180 rpm allows the particles to attain a sufficiently large size (diameter of about 1 *μ*m in this case). The birefringent particles are ingested by the cells via the phenomenon of endocytosis.

A schematic diagram is shown in Figure 1A. A 1064 nm Gaussian beam in TEM_00_ mode from a diode laser is used for trapping. A polarising beam splitter (PBS 1 in Figure 1A) is used to split the incident beam into two orthogonally polarized components. In our experiment we choose the p-polarization. The dichroic mirror reflects and directs this polarized 1,064 nm trapping beam towards an oil immersion objective lens (×100 Olympus, 1.3 NA). The objective focuses the trapping beam on the sample chamber. The forward scattered light along with unscattered light is collected by an air immersion condenser lens (10× Nikon, .25 NA). This forward scattered light is then reflected by another dichroic mirror towards another polarizing beam splitter (PBS 2) which is used to direct the p-polarized component of the mixture of forward scattered and unscattered beam towards a quadrant photodiode (QPD). The scatter pattern for the birefringent particle while turning in the pitch sense under crossed polarizers is also shown in Figure 1, as indicated in [30]. The difference in halves of the scatter pattern is used to ascertain the pitch rotational angle of the particle, while trapped inside the cytoplasm. We would like to reiterate here that since the particle is optically trapped with a linearly polarized light, the particle is orientationally confined to initially be along the direction of polarization. It then executed thermal motion about the direction of polarization.

The interference between the scattered and unscattered light forms a scatter pattern on the QPD [31, 32]. Cross polarizers effect is brought about by a half wave plate (HWP) placed in the path of the outgoing laser beam before the second PBS. The HWP is used to fix any complicated phase changes in the outcoming light. It is so adjusted that in absence of any particle, the light orthogonal to incident polarization is minimum in intensity. Any motion of the trapped particle causes a difference in illumination between the halves of the QPD. The difference between right and left halves of the QPD gives a measure of displacement along *X*-axis and that between the upper and lower halves give a measure of the displacement along *Y*-axis. Any displacement along *Z*-axis (direction of propagation of light) causes a change in total illumination of the QPD. This signal passes from the detector of bandwidth 40 KHz to a computer using data acquisition cards (DAQ, National Instruments, NI PCI 6143) having a bandwidth of 40 KHz.

Besides translation, the particle has three degrees of rotation. We probe the out of plane rotation in pitch degree of freedom as in Figure 1B. For pitch detection, a pair of photodiodes is used instead of a QPD. The s-polarized component of the beam transmitted from the other port of the PBS is made to fall on an edge mirror which splits the light into two halves A and B (Figure 1C), each half of which is made to fall on a photodiode (A and B) (Figure 1A). Signals from the photodiodes are connected to amplifers and then to the computer *via* DAQ. Rotation in the pitch sense results in a difference in illumination of the photodiodes as shown in Figure 1C) [32–34]. The experiment is conducted at a constant temperature of 25 C by keeping the room temperature constant with an air conditioner. The power of the 1,064 nm laser beam is about 60 mW at the sample plane for avoiding damage to the cell due to heating. Power spectral densities are recorded for trapped RM-257 particles inside the cells at a sampling rate of 40 kHz and averaged every 2 s. Recorded average over 10 such PSDs are used for viscoelasticity measurements.

## 3 Theory

We fit the power spectral density of the thermally driven fluctuations of the position and orientation of a particle optically trapped inside a cell with the Generalised Maxwell’s model or the Jeffery’s model [20–22]. That is assumed to have a solvent (the viscous part) with dissolved solutes (like polymers to give it elastic behavior) in it. The frequency-dependent viscosity in an incompressible low Reynolds number general linear viscoelastic fluid medium comprising of a solvent and a polymer solute dissolved in it is found to be given by the expression derived from the Stokes Oldroyd-B model for linear microscopic viscoelasticity [16, 35].

(1) $$\mu (\omega )=\mu_{s}+\frac{\mu_{p}}{-i\omega \lambda +1}$$

where, the *μ_s_* is the zero frequency solvent viscosity, *μ*_p_ is zero frequency polymer viscosity and *λ* is the polymer relaxation time.

This expression is similar to the Jeffery’s model or the Generalised Maxwell Model of frequency dependent viscosity with the coefficients labelled differently. Thus the viscosity of the solution at zero frequency would then be, *μ*_0_ = *μ_s_*+*μ_p_*. Solving for the power spectral density (PSD) of the optically trapped particle in this viscoelastic fluid, we get [16].

(2) $$PSD(\omega )=\frac{2k_{B}T}{\gamma_{0}}\frac{\left(\frac{\left(1+\frac{\mu_{p}}{\mu_{s}}\right)}{\lambda^{2}}+\omega^{2}\right)}{\left[{\left(\frac{\kappa }{\gamma_{0}\lambda }-\omega^{2}\right)}^{2}+\omega^{2}{\left(\frac{\kappa }{\gamma_{0}}+\frac{1}{\lambda }\left(1+\frac{\mu_{p}}{\mu_{s}}\right)\right)}^{2}\right]}$$

Here, the term *κ* signifies the trap stiffness and *γ*_0_ is the drag coefficient for only the solvent. The exact expression for the *γ*_0_ is then given by,

(3) $$\gamma_{0}=6\pi \mu_{s}a_{0}$$

where, a_0_ is the radius of the spherical particle. In order to correlate with the experimentally obtained power spectral density curves for the translational and rotational motion, we rewrite Eq. (2), as [16].

(4) $$PSD(\omega )=y_{0}+\beta^{2}A\frac{\left(\frac{\left(1+\frac{\mu_{p}}{\mu_{s}}\right)}{\lambda^{2}}+\omega^{2}\right)}{\left[{\left(\frac{\kappa }{\gamma_{0}\lambda }-\omega^{2}\right)}^{2}+\omega^{2}{\left(\frac{\kappa }{\gamma_{0}}+\frac{1}{\lambda }\left(1+\frac{\mu_{p}}{\mu_{s}}\right)\right)}^{2}\right]}$$

where the coefficient A indicates the amplitude in terms of Volts2/Hz and the calibration factor is then *β* in (m/Volt) quite akin to the conventional calibration factor for normal media [36]. From this fit, 4 parameters can be extracted, namely, $1+\frac{\mu_{p}}{\mu_{s}},\lambda ,\frac{\kappa }{\gamma_{0}}$ and *β*2A [12]. The y_0_ constant is also added to the power spectra density to account for the system noise floor. We use this expression for the PSD to understand the rheological properties of the intracellular cytoplasm, as a viscoelastic fluid. In this paper, we do not try to estimate the value of the calibration factor, thereby allowing the PSD to be normalised to 1. Then we only have 3 parameters to extract from the fit to the PSD. We assume that the solvent is water, such that *μ_s_* is that for water. Moreover, the *γ*_0_ is the drag in solvent which is water [12]. The power spectral density of the two different degrees of freedom are found and matched with the theoretical formula (Eq. 4). This formula has been calculated in [16], as a solution to the Langevin equation where the viscosity has been made frequency dependent (to show viscoelasticity) in accordance with the Jeffery’s model.

The model can be further improved by adding active forces. There have been many attempts at showing that the effective Langevin descriptions in terms of friction memory kernels and additive active forces naturally emerge for models of confined Brownian tracers interacting with active suspensions [37–40]. This would be a direction of research that future models would explore, which however, is beyond the scope of the present manuscript.

In order to compute the G’ and G”, we use the relations

(5) G”=ωRe(μ)

(6) $$G^{\prime }=\omega Im(\mu )$$

These equations were borrowed from [41], where a detailed comparison was made with this method of computing G’ and G” with other methods.

The calibration factor for the translational signal is related to the temperature as

(7) $$\beta^{2}A=\frac{2k_{B}T}{\gamma_{0}}$$

Thus, the calibration factor *β* is then given as

(8) $$\beta =\sqrt{\frac{2k_{B}T}{A\gamma_{0}}}$$

Fitting the power spectral density with this Eq. (4), we can extract the values for the relative viscosity $\left(\frac{\mu_{s}+\mu_{p}}{\mu_{s}}\right)$ of the solution, polymer relaxation time constant(*λ*), the trap stiffness *κ* and the calibration factor *β*. The calibration factor only indicates how to convert between the V2/Hz to nm2/Hz and thus, to reduce the number of fitting parameters, we could normalise the curve to the maximum to find all other parameters and then come back to this parameter later.

## 4 Results and discussions

**Figure 2:**
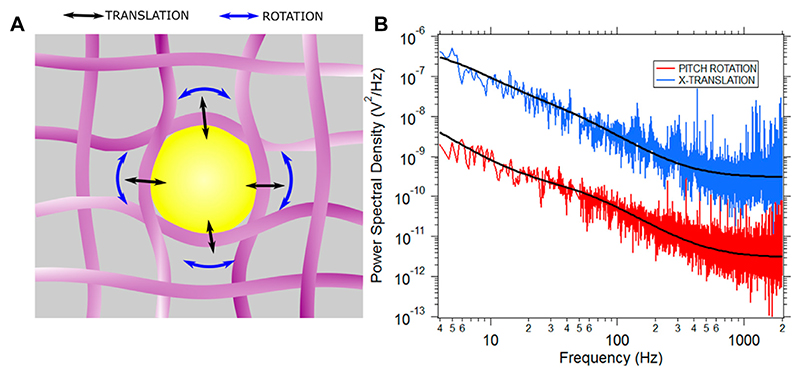
**(A)** A particle in the cytoplasm gets enmeshed in the meshlike network of cytoskeletal fibres. The cartoon is not to scale and is for representational purposes only.**(B)** Typical PSD for X(translation) and pitch (rotation) motion is shown in blue and red respectively. Both PSDs are fitted with the Jeffery’s model for viscoelasticity which yields the values $\frac{\mu_{\rho }}{\mu_{s}}+1=3\;\pm \;1$, *λ* = .009 ± .002 s for translational mode, and $\frac{\mu_{p}}{\mu_{s}}+1=2\;\;\pm \;\;1$, *λ* = .007 ± .002 s for pitch rotational mode as the fitting parameters.

The cell cytoplasm is a poroelastic medium [42, 43]. Here, there is a cytosolic fluid in a cytoskeletal mesh comprising of microtubules, actin filaments and intermediate filaments like vimentin [5, 44–46]. If a particle larger than 100 nm radius is placed inside the cell, trapped and PSD recorded, one can believe the case to be that of Figure 2A [17, 44, 47].

**Figure 3:**
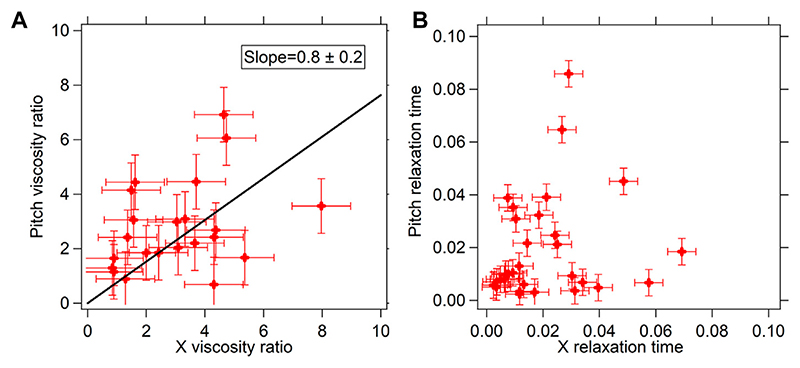
**(A)** Viscosity ratios $\left(\frac{\mu_{p}}{\mu_{s}}+1\right)$ for pitch (rotational) motion are shown as a function of corresponding viscosity ratios for X (translation) motion **(B)** Relaxation time (*λ*) for pitch (rotational) motion are shown as a function of corresponding viscosity ratios for X (translation) motion. Black solid lines in **(A)** shows linear fit to the scattered plot but assumed to pass through the origin.

The translational mode leads to stretching of the cytoskeletal fibers in a different fashion than that of the rotational modes. Thus, if the PSD reflects the cytoskeletal modes, the rotational and translational PSD should bear different parameters [24]. We go on to study the translational PSD and the rotational PSD. A set of typical curves are shown in Figure 2B. The experiments were repeated 31 times for birefringent particles trapped in optical tweezers. There were a total of 31 different cells, each data point being a particle optically confined in a different cell. The translational (*X*-axis) viscosity ratio of the cytoplasm to that of water and relaxation time was plotted as a function of the rotational pitch viscosity ratio and relaxation time performed in MCF-7 cells, shown in Figure 3.

We find that at low 0 Hz viscosity, which appears away from the nucleus mainly, the values for translation and rotation are similar but at high values of viscosity, which appears close to the nucleus, there seem to be discrepancies which could be a result of the different modes of the cytoskeletal fibers being involved. There could be other effects involved too requiring more detailed study. Thus, we limit ourselves to measurements where both the viscosities are less than about 6 times that of water, as reported in Figure 3A. This comparative plot can be fitted well with a straight line of slope .8 ± .2, when assumed to pass through 0. We find that these X and pitch 0 Hz viscosity parameters match well with each other to a two-tailed *p*-value of .0014, indicated by a straight line fit passing through the data (which has been assumed to be passing through 0 since when the fluid has 0 mPa s viscosity for X, it should also have 0 mPa s viscosity for pitch). The straight line slopes are consistent with 1, within the error bars. We would also like to point out that such a comparison of the translational and rotational modes has never been performed in past. We would like to point out that the shape of the particles do indeed deviate from spherical to about 20% of the radius, as has been indicated in [27]. This can explain the differences in values obtained, as the rotational and translational drag forces would affect the system differently in the different modes. Moreover, since we are only looking at the passive thermal motion, it should ideally be uncorrelated in each degree of freedom, such that complex multiple degree-of-freedom rotation may not affect the thermal pitch rotation. Further higher resolution study is required to distinguish between the modes.

It has been stated in [12] that the values of the 0 Hz viscosity obtained for the cell cytoplasm in these experiments are between 1 and 15 times that of water, which is what one can obtain by dissolving such concentrations of intracellular proteins in water [48, 49]. It may be noted here that a solution comprising of a solvent, like water, with dissolved substances (can be called solutes) can indeed have a higher viscosity. That is the inherent assumption which we use to fit the model to the power spectral density inside the cell cytoplasm. Viscosity inside the cell varies from one location to the other and so does the refractive index [12, 14]. Probing viscoelasticity with the rotational mode can be advantageous in the sense that the particle does not actually move from one position to another but freely rotates at a particular location.

**Figure 4:**
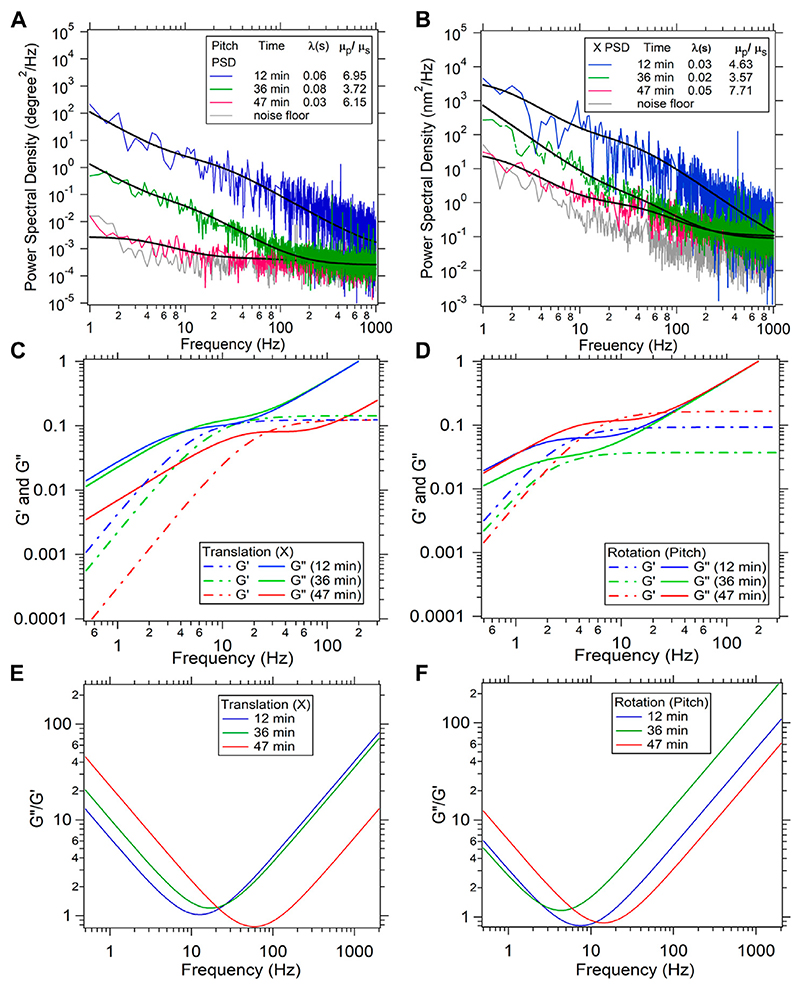
A time dependent measurement of viscosity ratio $\frac{\mu_{\rho }}{\mu_{s}}$ and relaxation time λ for translation(X) **(A)** and corresponding pitch (rotation) **(B)** of the same particle at same instant of time is shown. Time mentioned here is the duration since start of the experiment, when the healthy cell is placed onto the stage at 25 C room temperature. Fitto Eq. 4 is shown with black solid lines. **(C)** and **(D)** Storage modulus (G’) and loss modulus (G”) for X and Pitch for the three different PSDs is shown for translation and rotation **(E,F)**.

The discrepancies of the viscoelasticity at high 0 Hz viscosity could have other effects involved too. There could be variations due to the method with which the particles have been inserted into the cell. We incubate the particles with the cells as we mentioned in the materials and methods. This can introduce variations in the way the particle is lodged inside the cytoskeletal mesh, since the coatings on the particle due to the endocytosis can vary. One way of solving this problem is to use microinjectors to insert the particle, which can be performed in future experiments. We would also like to mention another facet of the rheology of the cells using our method. The same particle placed inside the cell at the same location can yield a changing 0 Hz viscosity as a function of time, as reported in Figure 4, Initially, in Figures 4A, B, which correspond to the two modes of motion of the particle, both the PSD’s start with high amplitudes but start to get lower towards the noise floor as a function of time, thereby indicating increase in the 0 Hz viscosity of the cytoplasm as a function of time. We speculate that the health of the cell is changing as a function of time but the exact mechanism would require more detailed study of the process. We also show the G’ and G” computed from the translation and rotation mode as a function of frequency in Figures 4C, D. It is evident that as the cell ages, the cytoplasm becomes more elastic than viscous, as the separation between the G” and G’ reduces. The cell can be called more viscous if the G” is higher than the G’, while it can be said more elastic in the reverse case. We find that G’ crosses the G” as the cell ages, which implies that it becomes more elastic than viscous during the process of aging.

This phenomenon could be a result of more crosslinks of the fibers forming inside the cell. It could also be the cytosol becoming more viscous due to depolymerization of the cytoskeletal fibers back into the aqueous solution.

We have also included a plot of the ratio of G” and G’ as a function of frequency in Figures 4E, F. We find that the frequency at which the ratio of G” and G’ is the lowest increases from freshly taken cells to aged cells. We also find that G’ saturates at higher frequencies which would imply that the extent of elasticity of the cytoplasm does not change beyond a certain frequency.

## 5 Conclusion

Thus, in conclusion, we find that the passive thermal motion of an optically trapped bead in the cell cytoplasm provides the viscoelasticity information of the cytoplasm, both in the rotational mode and the translational mode. When the 0 Hz viscosity values are low, particular away from the nucleus, the rotational and translational values are consistent to a *p*-value of .0014. However, many discrepancies appear when the 0 Hz viscosity values become large, possibly due to the involvement of modes of the cytoskeleton. More detailed study is required for exploring the discrepancies at higher values.

## Data Availability

The raw data supporting the conclusion of this article will be made available by the authors, without undue reservation.
